# The Teeth and Their Preservation

**Published:** 1876-06

**Authors:** Marshall H. Webb


AMERICAN JOURNAL

OF

DENTAL SCIENCE.

Vol. X. THIRD SERIES—JUNE, 1876. No. 2.

### ARTICLE I.

### *The Teeth and Their Preservation.*

BY MARSHALL H. WEBB, D. D. S.

Read before Wisconsin State Dental Society.

The natural teeth are not specially important whilst associate parts are in repose, but when the functions of the cerebrum are no longer suspended,—when the mental faculties are fully active, thus demanding a greater supply of arterial blood,—and some happy thought flits through the brain, the eye indicates it, a smile ensues and the teeth are shown, and if they are in quite perfect condition, then beauty asserts its presence even if the other features are not so beautifully proportioned as a form which Michael Angelo would conceive to be imprisoned in a block of marble, and, by the aid of hammer and chisel, set such form free.

When, however, the teeth are uncleansed, and a fungus, the *Protococcus dentalis*, collects upon the labial surface, or as a result of caries they are fractured, or the decay presents an opaque appearance through the translucent enamel then the beauty of facial expression is marred.

Culture ensues when one mingles in good society, but when the very soul is chained through the consciousness of this condition of those organs (which organs should give to facial expression one of its chief ornaments) an effort is made on the part of the individual to conceal such teeth, or, indeed this may lead the person, otherwise beautiful, and especially so, if possessed of true intelligence, to avoid the social circle altogether.

Now, in consideration of all this, those who operate upon the teeth cannot but confess, that, as the lapidist exhausts his skill in fashioning the native diamond into a specimen of beauty to add to personal charms, so, also, should they, pursuiug a higher and nobler calling, do their utmost to restore to a proper condition the priceless jewels given by natnre to constitute the most precious diadem which she has set within her choicest frontispiece.

Not only is it the duty of the operator to bring forth the pearl-like organs to their original beauty, comeliness and utility, but it is also necessary that he be qualified to give that advice which will enable the patient to keep the whole system in harmonious action. He should be capable of directing the observance of hygienic law through childhood and advancing years,—nay, through the mother, during the embryonic period and infancy. Nature will then avail herself of the opportunity to fully develop the various organs, and be the better enabled to endow the tissues with a due proportion of elements. When the organs are presented for treatment, one should be prepared to aid nature in the cure of disease in every phase, so far as it relates to the teeth and associate parts.

This implies that all which contributes to the qualifications of one as a successful general surgeon, also applies to one who devotes himself to special practice, but whilst he who applies himself to the special department of surgery should be skillful, as well as have a thorough understanding of the parts involved, yet he who devotes his time to dental practice should do more than this: he should perform all operations upon the natural teeth with what may be designated *artistic* skill. Caries of dental tissue must be removed and that which is lost must be substituted by the practitioner alone. Nature's form doth strength with beauty blend, and when no interruption occurs, all parts are arranged in beautiful symmetry of proportion throughout. If the surgeon fails to operate or arrange the parts operated upon in accordance with the designs of nature, then the typal presence resident within and about the cells which reconstruct the part, will yet be true to such design and do the work assigned them; but, when we come to the practice of the dental department of science, we find that whilst the oral surgeon should possess a general medical education if he be successful in the discharge of his duties in treating diseases of the teeth and associate parts; yet, in carrying into practice the principles of this department of science, it is, also, necessary for one, when substituting a material for dental tissue, which has been destroyed by caries, to perform the operation in an artistic manner and to follow out the beautiful natural type.

When nothing interferes with the natural processes, the cells—the odontoblasts—build up perfect tissues; the dental organs are properly formed and erupted, and second dentition follows the first in strict conformity with established laws. Often, however, it is necessary for the practiioner to observe dentition throughout its different phases, and to remove obstructions; to avert, modify or arrest irregularity of the teeth.

The deciduous teeth should be retained until displaced by encroachment of the permanent ones, though it is sometimes necessary to remove disintegrated tissue and fill cavities of decay in them that they may be preserved. The deciduous teeth should be extracted, however, when chronic abscess has taken place, thus averting an abnormal position of the permanent teeth. As they are dislodged and the permanent erupt, an examination should be made in reference to caries in the fissures of molars and bicuspids, and to disintegration of enamel upon the proximate surfaces. Often the enamel cusps do not coalesce in the sulci, and deep cavities are presented, though they may not be seen by unaided vision. If a section be prepared from that portion of a tooth, and placed under the microscope, those crevices can be distinctly seen. During mastication particles of food are impacted into these fissures, acid products are formed, disintegration ensues; destruction of dentinal tissue seems to be accellerated by the presence of *Leptothrix buccalis*, and very soon cavities of decay are plainly visible.

Alimentary substances lodge or become impacted between the teeth, acid formations result, and disintegration of enamel ensues. This may be prevented by polishing the proximate surfaces of the teeth frequently, and by having the patient pass floss silk or use the tooth pick daily between them. This will not require any further precautionary measures or more frequent visits to the office than would be necessary, if permanent separations were made.

Where disintegration of enamel takes place upon a proximate surface, and the operator is *positive* that it is only superficial, such tissue should be removed and an evenly polished surface carefully made. If the surfaces be left unpolished, disintegration may take place to an extent, even greater than before.

The disintegration can often be removed by means of a corrundum disk, without previous separation by pressure, but it is best to thus separate, to avoid needless cutting away of surrounding tissue, both from the surface upon which the disintegration is found, and from the proximate surface of the adjoining tooth. When the disintegration has been thus removed, the rubber dam should be applied to the tooth operated upon, as well as the one or more adjoining, and after they are dry, a careful examination should be made. If all the disintegration has been removed, the whole surface should present the appearance of normal tissue.

The surface operated upon should then be thoroughly polished; first, with strips cut from emery cloth of no. half and no. ought, and, after the removal of the rubber dam, with pulverized pumice, mounted upon linen tape, or applied upon a soft rubber disk. If, however, upon said examination, it be found that a circumscribed portion of enamel, having a chalky appearance, yet remains upon such proximate surface, then one can rest assured that the operation of removing the disintegrated tissue has not been successful, and that upon further examination with an instrument, it will nearly always be found that the disintegration extends to the dentine, thus necessitating the cutting out of all such decay, and the careful and thorough filling of the cavity presented.

Unless the progress of caries be arrested by artificial means, the work of decalcification goes on ; *Leptothrix* seem to accellerate the destruction of dentine ; pulp tissue is soon encroached upon, and through irritation it becomes inflamed and is destroyed. Indeed to caries may be traced almost every manifestation of disease connected with the teeth. Necrosis of the maxillæ may ensue because of the continuance of the pathological phenomenon known as chronic alveolar abscess ; mephitic gas arising from putrescent pulp tissue, and escaping through the apicial foramen, issues in alveolar abscess ; as a result of irritants finding lodgment in a cavity of decay, irritation, congestion, inflammation and devitalization of the pulp ensues, accompanied by various phases of odontolgia—and all this concomitant with dental caries.

When disintegration has taken place in a sulcus, or the sulci of a molar or bicuspid, the rubber dam should be neatly applied, so that the parts are made dry and brought more plainly to view, after which the cavity should be opened into by means of enamel chisels, (if caries has made considerable progress underneath the plate of enamel,) the softer portions of carious dentine should be removed by sharp excavators, and the fissures cut out by keen burs, when the margins of the cavity should be made regular, and sufficient anchorage secured for the filling, though but little undercut is necessary. The walls should be well shaped, the margins evenly and smoothly finished, a starting point made, and the cavity so prepared, that all the gold may be impacted by the aid of the mallet. If, in impacting gold, any part, or the whole, becomes displaced, it should at once be removed, otherwise the filling will be imperfect. All this is liable to occur where pallets, cylinders and similar forms of gold are used, though the movement of the gold may be so slight that the operator fails to notice it at the time. The gold should be so carefully prepared, introduced and impacted that one *knows* that each piece is firmly anchored, or has adhered to that already introduced, and being certain of this, the filling cannot but be solid and uniform in density. The gold should be so impacted as to be full with the margins yet made concave, and so as to correspond with the surrounding unimpaired tooth structure, although that part of the inclined portion of the cusps which is built out with gold, need not converge so as to form an acute angle as seen in nature.

When disintegration of enamel takes place upon the buccal surface of a molar or bicuspid, or the labial surface of the anterior teeth, and caries ensues so as to form a cavity, the operation of filling such a cavity is somewhat similar to that required where decay occurs in the fissures, with the exception, mainly, that moisture would interfere to a greater extent. Whilst desirable results cannot be obtained in the first mentioned class of cases, which are termed simple, (and which certainly are, when simply and indifferently treated,) but it is impossible to perform any but almost worthless operations where decay attacks the buccal and labial surfaces, unless the most valuable of all dental adjuncts—the rubber dam—be properly applied. When caries extends beneath the margin of the gum, it is often with some difficulty that this can be adjusted. In such difficult cases, the ligature—the waxed floss silk—should be placed twice around the tooth to be operated upon, after the rubber dam is securely adjusted to the adjoining teeth, and, whilst holding the ends of the ligature in the left hand, both the ligature and rubber dam should be pressed above the cavity which is then clearly presented. It is often necessary to hold the ligature, together with the rubber dam, above the margin of a cavity occuring in the labial portion of an anterior tooth, by means of a broad pointed excavator. Before commencing such an operation one should have everything at hand which may be required. A clamp should be used when adjusting the rubber dams to the lower molars.

After the removal of carious dentine by keen excavators, the margin of cavity should be made regular, and all the disintegrated enamel be removed by means of a sharp plug finishing burr, operated with a dental engine. There should be a slight undercut made all around the cavity at about the part where the enamel and dentine coalesce, and a point drilled in a direction where there will be no liability of approaching pulp tissue—this to serve as a starting point.

When small cavities are to be filled, a half leaf of light foil should be taken from the book by means of the foil carriers, and placed upon a piece of spunk, mounted upon a small, thin, block of wood, covered by white kid. This should be folded with an ivory spatula into a ribbon-like form of about three lines in width, and then taken in the foil carriers and cut into parts one and two lines wide. These parts, as they are cut, should fall upon spunk which has been covered by kid, from which they can be readily taken up previous to introducing into the cavity with the plugging instrument. When spunk is covered with kid for folding the gold upon and for picking it up from, one avoids the liability of occasionally introducing spores from ths spunk in connection with the gold.

When large fillings are to be inserted, a whole leaf of light foil should be folded and cut in the manner described, even more may be thus prepared. Heavy foils should not be undervalued, especially where restoration of contour is required to a considerable extent, although light foil, prepared as just described, and not brought in contact with the fingers or with moisture, is more readily impacted into the irregularities, undercuts, and all such parts where it may be necessary to do this by hard pressure. When this is done, and anything approximating direct force can be applied, a suitable plugging instrument, having finely serrated points, should be selected, placed in position in the electro-magnetic mallet, and, by the aid of this instrument, each piece of gold should be perfectly impacted. The gold should now be taken upon the point of the pluggeing instrument, and passed through the flame of alcohol, though it should not be subjected to more heat than to bring about a rose-tinted hue—this to cause the gold to cohere more quickly, and the cohesion of particles to be more perfect. When an operator has not the ability to perform, or a patient cannot really afford first-class operations, then it may appear necessary to insert tin foil, Hill's stopping, oxychloride of zinc, or, as a last resort, and in preference to extraction, amalgam.

Many practitioners take gold or tin-foil, and roll it into a shapeless mass and bring it into direct contact with their fingers, or they mix up amalgam, and then place one of these materials into the sulci of molars or bicuspids in an indifferent manner, and all this, too, in cavities only partially and imperfectly prepared. At the same time they may have been fully aware that the proximate surfaces were also involved. In due time carious action so far advances as to involve the dentine immediately overlying pulp tissue and to cause odontalgia. The patient again calls and is informed that the tooth must be extracted before relief can be afforded, and thus, in a short time, the *mal*-practitioner brings about an edentulous condition, thus making it necessary to insert an artificial denture. Among this class we find those who talk most about "*educating the people*," and of "*compensation.*" and who complain of "*non-recognition*" by medical gentlemen. It is truly a wonder that the people have any confidence in dental operations at all, and especi ally when they are called upon to lose, not only the amount paid for such operations as are of little or no value, but the priceless pearls given them by nature, whilst he who is guilty of such mal-practice does not deserve recognition.

When disintegration of enamel takes place upon the approximating surfaces of the teeth, and caries attacks the dentinal structure, then the operation of removing the disintegrated tissue and filling the cavity thus formed is necessitated. This operation is more difficult than those described and but few operators perform it well.

Permanent separation is sometimes made with the object of getting at the part affected, and thus a portion of good tooth structure is needlessly sacrificed. Such operations are often performed so that the irregular and roughened surface of enamel, and perhaps portions of dentine remain exposed. Food passes along the inclined surface to the neck of the tooth, and is there impacted between it and the one with which it comes in contact, and acid products are formed. If the tooth structure does not soon become more dense, or the patient is not very careful in keeping the parts not only well cleansed, but endeavors to gradually polish the surface, disintegration will ensue to a *greater* extent than before. Where care is taken, the teeth so separated and the approximate surfaces so shaped that food will not be so liable to become impacted at the necks of the teeth, and the whole operation of removing all disintegrated tissue, forming the cavity, inserting the filling and polishing the surface is thoroughly performed, caries will not so readily recur; even when such operations are thus carefully performed, they are not the most desirable, although a greater number of these can thus be performed within a given time, and those who aim to make the most of the time during which they operate, *may be successful—financially*,—but the time is coming when patients will protest against all this, and when the services of those who are faithful because of an innate consciousness, rather than that such faithfulness depends upon the fee they expect to receive, shall be appreciated more and more, and, coming down to a secondary consideration, all such faithful practitioners will find that the compensation will also be greater even and anon.

When the enamel of the proximate surfaces of the molars and bicuspids is penetrated and the dentine is involved in the carious process, and separation by pressure has been made and the rubber dam applied, the cavity should be opened into from the masticating surface, (excepting in rare cases when an opening through the buccal wall of enamel may appear necessary to reach a small cavity near the neck of the tooth,) for, even where but little structure *appears* to be impaired, this surface would be almost approached upon the removal of the disintegration, and this portion of the plate of enamel would be liable to fracture if allowed to remain. It is far better to cut away this bridge of enamel, than to needlessly sacrifice structure along the entire proximate surface; in addition to that, a better view can be obtained, and thus a more perfect removal of disintegrated tissue will be facilitated, and the gold can be more thoroughly impacted; hence, the operation can be more perfectly performed in this way than in any other manner. Such a cavity should generally be prepared in connection with the adjoining sulcus, or fissure, and the operations completed at the same time.

In the preparation of such a cavity, the edges of enamel should be made regular, and a groove be cut around the cavity near where the enamel and dentine coalesce, and especially should such groove be quite well marked along the buccal and palatal walls of enamel. A starting point should be made in that portion of the cavity which extends near the neck of the tooth, not in a direction where the pulp would, in any manner, be injured, but longitudinally and about where the dentine coalesces with the other hard tissue of the part—the enamel of the cementum. This point should not be deep but just sufficiently so, that when the small pieces of gold are introduced and impacted, they shall be retained, whilst other such pieces are being built upon this foundation.

The gold should not only be built against every part of the dentinal structure, but be impacted as perfectly as possible against, and to some extent over the margins of the enamel. To accomplish this, it is often necessary to build the gold against the proximate surface of the adjoining tooth, but if there be a similar cavity in such tooth, or too much space intervenes, a matrix, made of a rather smoothly worn 00 separating file, may be adjusted.

After the gold has been impacted piece by piece, so that the substitution for lost tissue is complete, a 00 separating file should be used to cut away the surplus gold, and to assist in giving the filling a proper shape, which shape should conform to the original contour of the teeth. Strips cut from emery cloth of the finest grades, should now be used, which emery makes the surface regular and quite smooth, and leaves the parts gracefully formed. All this must be done before the rubber dam is removed, after which the finishing should be completed by the use of finely pulverized pumice or silex mounted upon linen tape. If the application of fine rouge to parts about the mouth be not considered objectionable, a small portion may be applied upon a strip cut from fine chamois skin. This leaves a more desirable finish than the burnisher. The finishing of the gold upon the masticating surface should be done with corrundum cones and Hindoostan stones, followed by pumice, mounted upon rubber, wood or leather points, after which, the burnisher may be used.

When disintegration of enamel takes place upon the proximate surface of the anterior teeth, separation by pressure is necessary. Such separation should be effected by means of wood or cotton placed between the teeth. After some space has thus been gained, it may be still further enlarged by the filling and wedging in of Hill's stopping. Whatever irritation may have ensued in consequence of the wedging will then rapidly pass away. Under no circumstances should rubber be made use of for this purpose. Quick wedging is occasionally resorted to, and at times may be necessary.

When sufficient space has been obtained, the rubber dam should be neatly and properly applied and the operation commenced. Here, as elsewhere, the cavity should be properly prepared, as before indicated, and the operation so completed that there shall be a perfect restoration of contour. All operations should be performed as perfectly and artistically as possible, so that if gold is exposed it shall be attractive rather than repulsive to the eye.

Disintegration of enamel upon the approximating surfaces of the teeth commences almost invariably at, or about the point, or points of contact, and, when the dentine is reached, caries ensues beneath the enamel in all directions.

In the performance of operations upon the teeth thus affected, even if the decay has advanced to the neck of the tooth, or whether the carious process be ever so extensive, the contour should be so restored that when the teeth approximate, the gold shall come in contact with the adjoining tooth, if the structure be yet normal, or with the filling, if one has been inserted. Operations should be thus performed whenever disintegration of enamel takes place upon the proximate surfaces, and the dentine is involved, and where such carious process has resulted in still greater destruction of tissue and fracture ef enamel, thus necessitating more extensive restoration of contour. Attenuated walls of enamel should be cut down until regular and firm margins are made, and so that, in the restoration of contour, the gold mav be built over the prepared edges of such walls, and thus protect them.

Were tooth structure always perfect ; general, or special functions always normal ; nutriment always rich and abundant ; the salivary, mucous and gastric secretions never vitiated ; then would a fungus be as harmless, the oral fluids be as advantageous, and the natural teeth acted upon in very much the same manner as the pebbles over which the fresh water of the brook flows. Whilst observing the rippling waters of the brook, however, we notice that, although the bubbling water, leaping over pebbles and larger obstructions, does keep the surface, upon which this friction occurs, constantly cleansed, yet where this friction does not take place to such an extent, or not at all, we find that a fungus collects. Especially is this the case when a stone, or a portion thereof, is, for a portion of the time at least, not subjected to the washing, thus affording lodgment for the fungus, *Pennicillium glaucum.*

Phenomena, somewhat of this character, take place in the oral cavity, although here, acid conditions exist and disastrous results ensue. That which most nearly resembles the phenomenon of the lodgment and proliferation of fungi upon stone, is the green-tinted fungus—the *Protoccoccus dentalis*—which collects upon the labial surface of the anterior teeth—this fungus having been regarded by M. Hallier as a form of development of the *Pennicillium glaucum.*

When caries ensues upon a proximate surface, so that a cavity is presented, and a permanent separation be made, a considerable portion of tooth-structure then becomes exposed. This surface affords lodgment for particles of food to a greater extent than before. Be that surface polished ever so thoroughly, it cannot be made as perfect as it was originally. Now, whilst the buccal and labial and the palatal and lingual surfaces are pretty thoroughly washed by the oral fluids, yet the proximate surfaces being out of the course of the direct current or movement of saliva, are but quite imperfectly kept free from particles of food and from *Leptothrix.* It is true that such surfaces *can* be kept clean; but this is more easily done where double V-shaped separations are not made, but where restoration of contour has been successfully accomplished ; besides, there is less exposed surface of tooth structure to be cleansed where contour operations have been properly performed. When the contour is restored, the surface approximating the adjoining tooth, being gold, cannot be acted upon by agents which bring about decay, whilst the margins of enamel, against which the gold is impacted, should not come in contact with the adjoining tooth at any point, and thus free, such remaining structure will be cleansed by the saliva, which is kept in almost constant motion by the action of the tongue, lips and cheeks, through the muscles of the parts.

Such fillings as described, when carefully and skillfully inserted, best subserve the purposes of mastication, prevent wedging of food against, and the consequent recession of the gums, protect the walls of enamel, and present an appearance at once inviting and beautiful. All such operations are difficult, however, but when one has the proper understanding of what they should be, and the ability to carry into execution the design, as furnished by nature, and then carefully, conscientiously, and thoroughly perform the same, they stand forth as monuments of skill, and are the best for the preservation of the remaining tooth structure.

